# Rapamycin mitigates neurotoxicity of fluoride and aluminum by activating autophagy through the AMPK/mTOR/ULK1 pathway in hippocampal neurons and NG108-15 cells

**DOI:** 10.1038/s41598-025-94648-0

**Published:** 2025-03-21

**Authors:** Dan Tao, Ya Xia, Qilong Liao, Xuemei Yang, Luwen Zhang, Chun Xie

**Affiliations:** 1 School of Public Health, the Key Laboratory of Environmental Pollution Monitoring and Disease Control, Ministry of Education, Guizhou Medical University, No.6 Ankang Road, Guian New Area, Guiyang, 561113 Guizhou People’s Republic of China; 2 Collaborative Innovation Center for Prevention and Control of Endemic and Ethnic Regional Diseases Co-constructed by the Province and Ministry, Guizhou Medical University, Guiyang, 561113 People’s Republic of China; 3 State Environmental Protection Key Laboratory of Environmental Pollution Health Risk Assessment, Research Center of Emerging Contaminants, South China Institute of Environmental Sciences, Ministry of Ecology and Environment, Guangzhou, 510655 People’s Republic of China

**Keywords:** Fluoride combined with aluminum, Autophagy, Hippocampus, Rapamycin, AMPK/mTOR/ULK1 signaling pathway, Biochemistry, Environmental sciences, Neurology

## Abstract

Our previous studies have confirmed that fluoride combined with aluminum (FA) can induce hippocampal neuron damage in the second-generation offspring (F2) of rats; however, the underlying mechanisms remain unclear. In this study, we established an F2 rat model and an NG108-15 cell model to investigate the potential modes of action. The autophagy of F2 rat hippocampal neurons and NG108-15 cells was assessed using transmission electron microscopy and immunofluorescence/immunocytochemistry kit, respectively. Hippocampal morphology was evaluated via hematoxylin-eosin (HE) staining. We measured mRNA levels of AMPK, mTOR, ULK1, and LC3 using quantitative reverse transcription PCR, and protein expressions were analyzed by Western blotting. Following treatment with rapamycin (Rap) in FA-exposed F2 rats and NG108-15 cells, a small number of primary lysosomes and autophagosomes appeared within hippocampal cells, with HE staining indicating a near-normal restoration of pyramidal cell morphology. The quantity, intensity, and volume of green fluorescent spots in the cytoplasm of NG108-15 cells increased as observed through fluorescence microscopy. The mRNA expressions of AMPK, ULK1, and LC3 were upregulated while mTOR expressions were downregulated in NG108-15 cells. Correspondingly, protein levels for AMPK, p-AMPK, ULK1, p-ULK1 along with the LC3-II/LC3-I ratio increased whereas those for mTOR, p-mTOR and p62 decreased significantly. Similar trends regarding both mRNA and protein expression were noted within the hippocampus of F2 rats as well. Activation of the AMPK/mTOR/ULK1 signaling pathway by Rap enhances FA-induced autophagy thereby mitigating neuronal damage.

**Supplementary Information:**

The online version contains supplementary material available at 10.1038/s41598-025-94648-0.

## Introduction

Fluorine (F) is an essential trace element for humans, characterized by a narrow safety threshold and high chemical reactivity. It can react with various elements to form fluoride at room temperature^1^. Industrial emissions, wastewater, and waste residues contain fluoride, which can be discharged into the environment, causing F pollution. While moderate F levels are beneficial for health, excessive intake can lead to bone damage, manifesting as dental and skeletal fluorosis. Additionally, it can induce injuries to multiple organ systems^2,3^. F exposure has been confirmed to cause structural abnormalities in neurons and damage to the hippocampus, resulting in central nervous system dysfunction^4^. Meanwhile, aluminum (Al) is the most abundant metal element in nature and is widely used in manufacturing, food processing, and production packaging^5^. Accumulation of aluminum in the body to toxic levels can cause nerve damage and cognitive disorders, such as Alzheimer’s disease (AD) and Parkinson’s disease (PD)^6–8^. Excessive Al exposure has been confirmed to lead to changes in neurobehavior and impairments in learning and memory abilities in rats^9^.

Epidemiological studies have found that pollutants discharged by electrolytic Al plants may increase the incidence of dental fluorosis among factory workers and surrounding residents^10^. And exposure to fluorine combined with aluminum (FA) can form more active complexes in the gastrointestinal tract, which accumulate in the brain, causing abnormal nerve cell morphology and pathological changes in brain tissue^11,12^. FA exposure has a severe impact on hippocampal neurons, significantly affecting the health, economy, and social development of exposed populations. Our previous study also indicated that continuous FA exposure damages the hippocampal neurons of offspring rats^13^. Thus, it is full of public health implications to elucidate the potential mechanism at the molecular level to provide a basis for scientific prevention and control.

Autophagy is a self-protection mechanism developed by cells through long-term evolution. In response to hunger, inflammation, and oxidative stress, autophagic lysosomes form to degrade organelles and misfolded proteins, regulate cell differentiation, rebuild tissues, support body development, and maintain homeostasis during disease^14–16^. During chronic hypoperfusion, autophagy degrades abnormal proteins and damaged organelles, provides energy for cells, and promotes damage repair, thus playing a neuroprotective role^17^. Microtubule-associated protein 1 light chain 3 (LC3) is a recognized biomarker of autophagy, integral to the entire autophagic process. There are two forms of LC3 in cells: LC3-I (cytoplasmic) and LC3-II (membranous). The LC3-II/LC3-I ratio generally reflects the level of autophagy^18^. Impaired autophagy has been reported to be crucial in F neurotoxicity^19^. P62 (also referred to as SQSTM1 or Sequestosome-1) maintains protein homeostasis by facilitating selective autophagy and is involved in various cellular signaling regulatory pathways and autophagic processes. During autophagy, p62 binds to ubiquitinated proteins and then forms a complex with LC3-II protein, which is localized on the inner membrane of autophagosomes, for degradation within autophagolysosomes^20^. P62 serves as one of the marker proteins reflecting autophagic activity, and its level indirectly indicates the clearance capacity of autophagosomes. Induction of autophagy can enhance the survival and functionality of cholinergic neurons, eliminate amyloid-beta and tau pathology, and improve memory in aluminum virus-associated Alzheimer’s disease nematode and rodent models^21^. Therefore, it is hypothesized that the mechanism underlying hippocampal neuron damage resulting from FA exposure may be associated with autophagy.

As a signaling molecule of autophagy, the mammalian target of rapamycin (mTOR) is involved in cell proliferation, differentiation, apoptosis, and autophagy by regulating protein synthesis and degradation^22,23^. Moreover, mTOR is closely related to learning and memory, and changes in mTOR activity can be detected in neurological diseases associated with hippocampal neuron damage^24^. During non-pathological aging, mTOR dysregulation may lead to impaired synaptic plasticity^25,26^. The AMP-activated protein kinase (AMPK)/mTOR/Unc-51-like kinase 1 (ULK1) signaling pathway regulates autophagy^27,28^. AMPK and mTOR participate in the development of neurodegenerative diseases during aging through autophagy^29–31^. These results suggest that the AMPK/mTOR/ULK1 signaling pathway may be related to hippocampal neuron damage. So far, there have been no reports on the relationship between FA-induced hippocampal neuron damage and the AMPK/mTOR/ULK1 signaling pathway.

Rapamycin (Rap) is an inducer of autophagy and the first known inhibitor of mTOR signaling, which effectively inhibits mTOR activity, thereby enhancing autophagy signaling^32^. Current studies have shown that inhibiting mTOR activity in cells can decrease the risk of various neurological disorders^33^. Rapamycin inhibition of mTOR expression alleviates nerve injury and movement disorders in mice, and reduces neurodegenerative changes in a mouse model^34^. Long-term low-dose oral Rapamycin can improve hippocampal neuronal damage in adult mice^35^. Therefore, it is speculated that Rapamycin therapy may have a neuroprotective effect on F2 rats exposed to FA.

Therefore, we established both the F2 rat model and the NG108-15 cell model of FA exposure with and without Rap treatment. We observed behavioral and hippocampal tissue structure changes in the F2 rats. Additionally, we measured the autophagy in the F2 rat hippocampus and NG108-15 cells, as well as the RNA and protein expression levels of AMPK, mTOR, ULK1, LC3 and p62. Furthermore, we investigated the influence of the AMPK/mTOR/ULK1 pathway on autophagy in FA-induced hippocampal damage and the role of Rapamycin in mitigating FA-induced hippocampal neuronal damage by affecting autophagy. The results of this study aim to provide a scientific understanding for the treatment of FA-induced hippocampal neuronal damage.

## Results

### The effect of Rap on the viability of NG108-15 cells

**Fig. 1:**
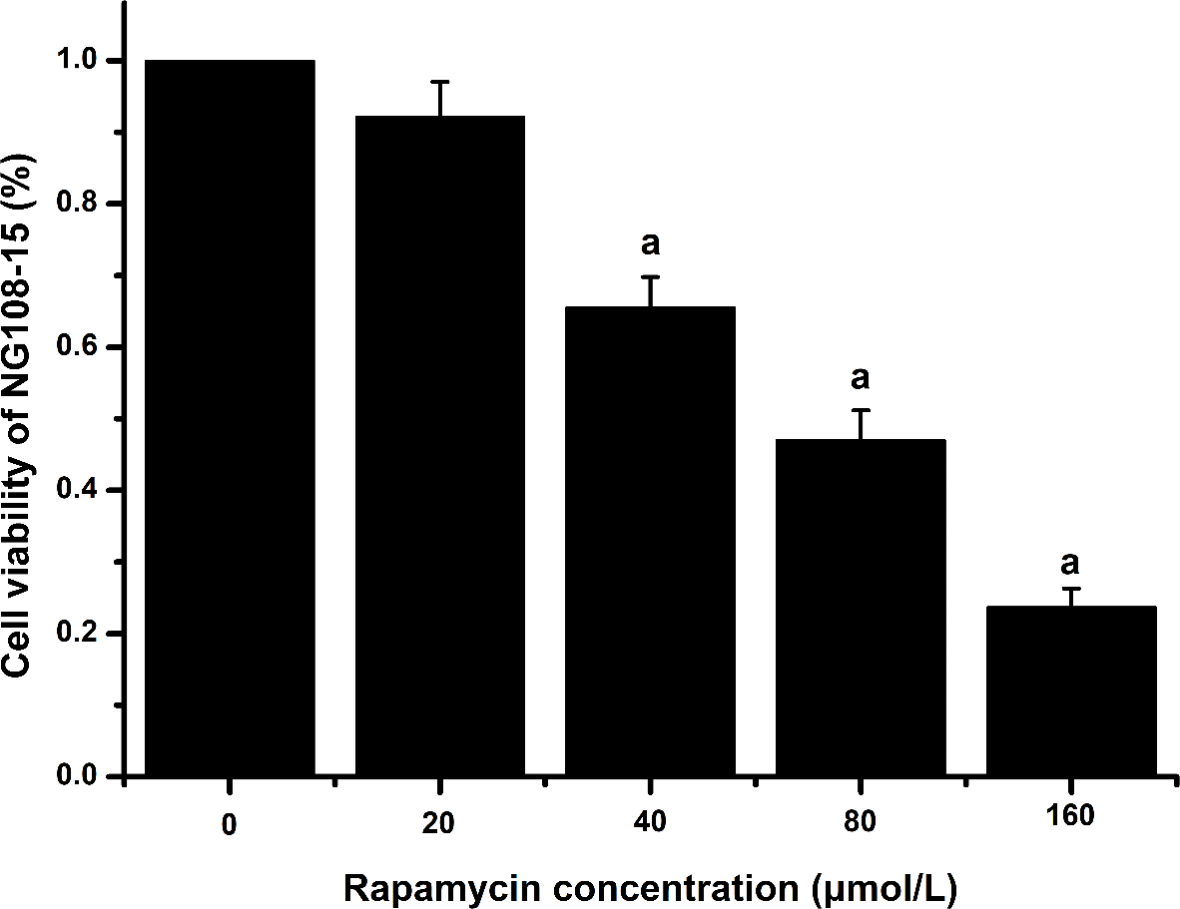
The effect of Rap on the cell viability of NG108-15. ^a^*P* < 0.05 versus the control group (*n* = 6).

The viability of NG108-15 cells treated with 40, 80, 160 µmol/L Rap was lower than that of the control group (*P*<0.05), shown in the Fig. 1. However, there was no significant difference in cell viability between the 20 µmol/L Rap group (92.16%) and the control group.

### The effect of Rap on the autophagy of NG108-15 cells

**Fig. 2:**
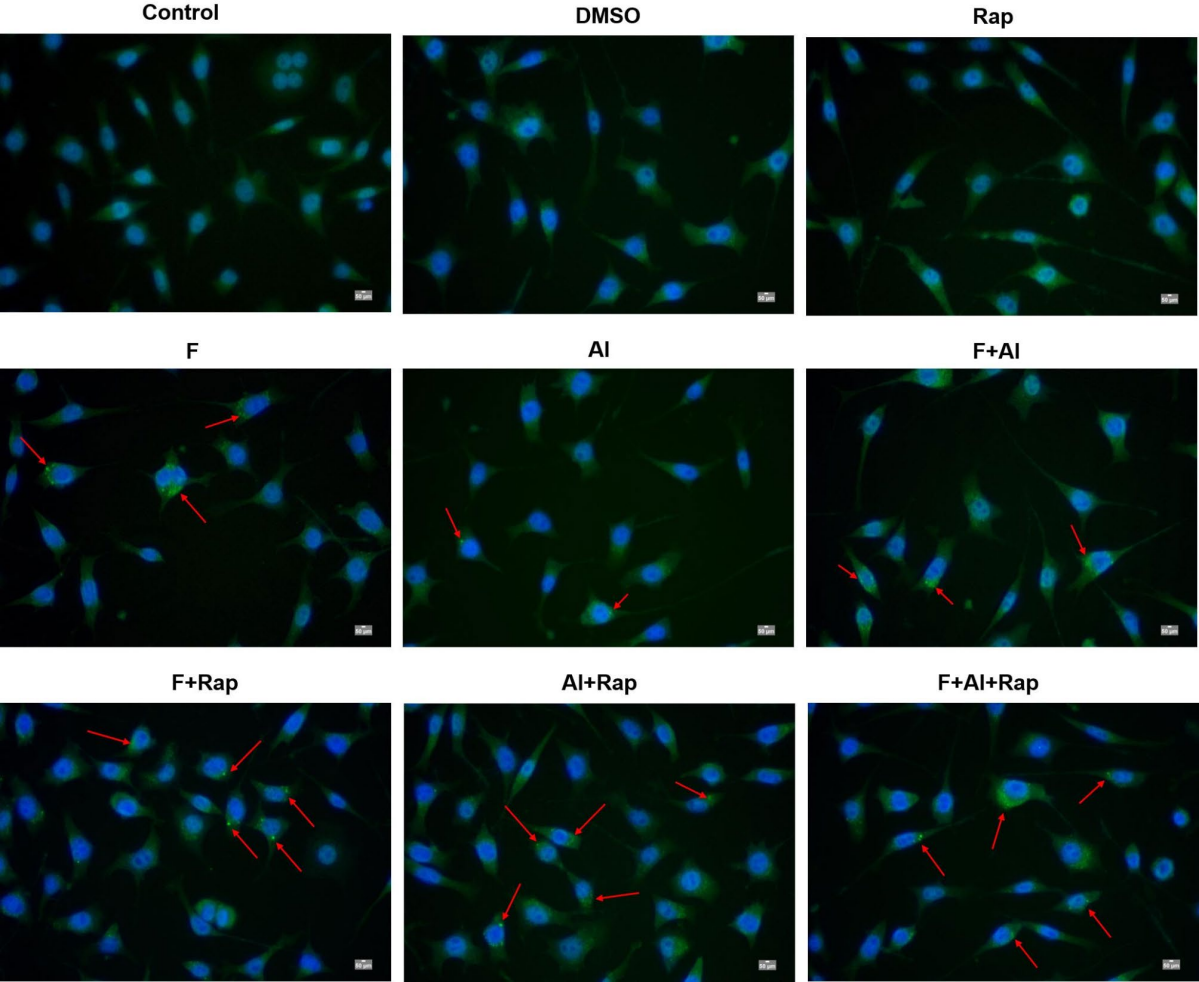
Fluorescence microscopy images of the autophagy in the NG108-15 cells (×200). The red arrow represents the autophagosome with green fluorescent spots.

The green fluorescent spots indicated the autophagy of NG108-15 cells. As shown in Fig. 2, no green fluorescent spots were observed in the cytoplasm of NG108-15 cells in the control, DMSO, and Rap groups. In contrast, green fluorescent spots were present in the cytoplasm of NG108-15 cells in the F, Al and F + Al groups. Compared to the DMSO group, the F + Rap, Al + Rap, and F + Al + Rap groups showed an increased intensity and volume of green fluorescent spots in the cytoplasm.

### The effects of Rap on mRNA and protein expression levels of AMPK/mTOR/ULK1 pathway and LC3 in NG108-15 cells

**Fig. 3:**
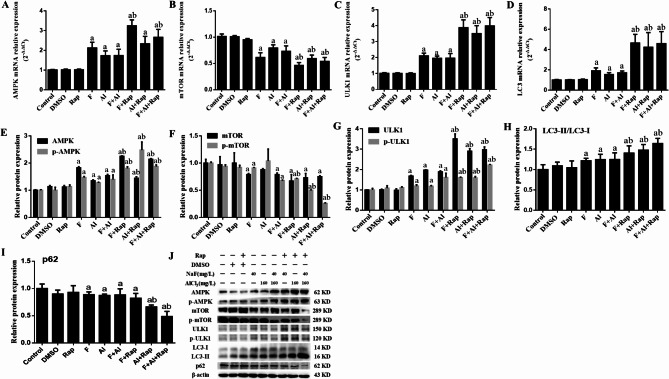
The mRNA expression (**A**–**D**), protein expression (**E**–**I**) levels and the original gel electrophoresis (**J**) of AMPK/mTOR/ULK1 pathway and, LC3 and p62 in NG108-15 cells. ^a^*P*<0.05 versus the control group, ^b^*P*<0.05 versus the exposure groups (n = 6).

As shown in Fig. 3A–D, the mRNA expressions of AMPK, ULK1, and LC3 increased in all experimental groups except for the DMSO and Rap groups (*P*<0.05), while the mRNA expression of mTOR decreased comparison to the control group (*P*<0.05). Compared with the non-Rap treated groups, the mRNA expression of AMPK increased in the F + Rap and F + Al + Rap groups (*P*<0.05). Additionally, the mRNA expression of mTOR decreased, and the mRNA expressions of ULK1 and LC3 increased in the F + Rap, Al + Rap, and F + Al + Rap groups (*P*<0.05).

The protein expression levels were shown in Fig. 3E–J. Compared to the control group, the protein expression of AMPK, p-AMPK, ULK1, p-ULK1, and the LC3-II/LC3-I ratio were higher in all experimental groups (*P*<0.05), the p62 was lower in all experimental groups(*P*<0.05). Except for the Al group, the protein expression of mTOR and p-mTOR decreased in other experimental groups (*P*<0.05). Compared to the F, Al and F + AL groups, an increase in the protein expression of AMPK, p-AMPK, ULK1, p-ULK1, and LC3-II/LC3-I ratio was observed in NG108-15 cells treated with rapamycin (*P*<0.05), while the protein expression of p-mTOR decreased in the F + Rap, Al + Rap, and F + Al + Rap groups (*P*<0.05). The protein expression of p62 also decreased in the Al + Rap, and F + Al + Rap groups (*P*<0.05).

### The effect of Rap on the body weight of F2 rats

**Fig. 4:**
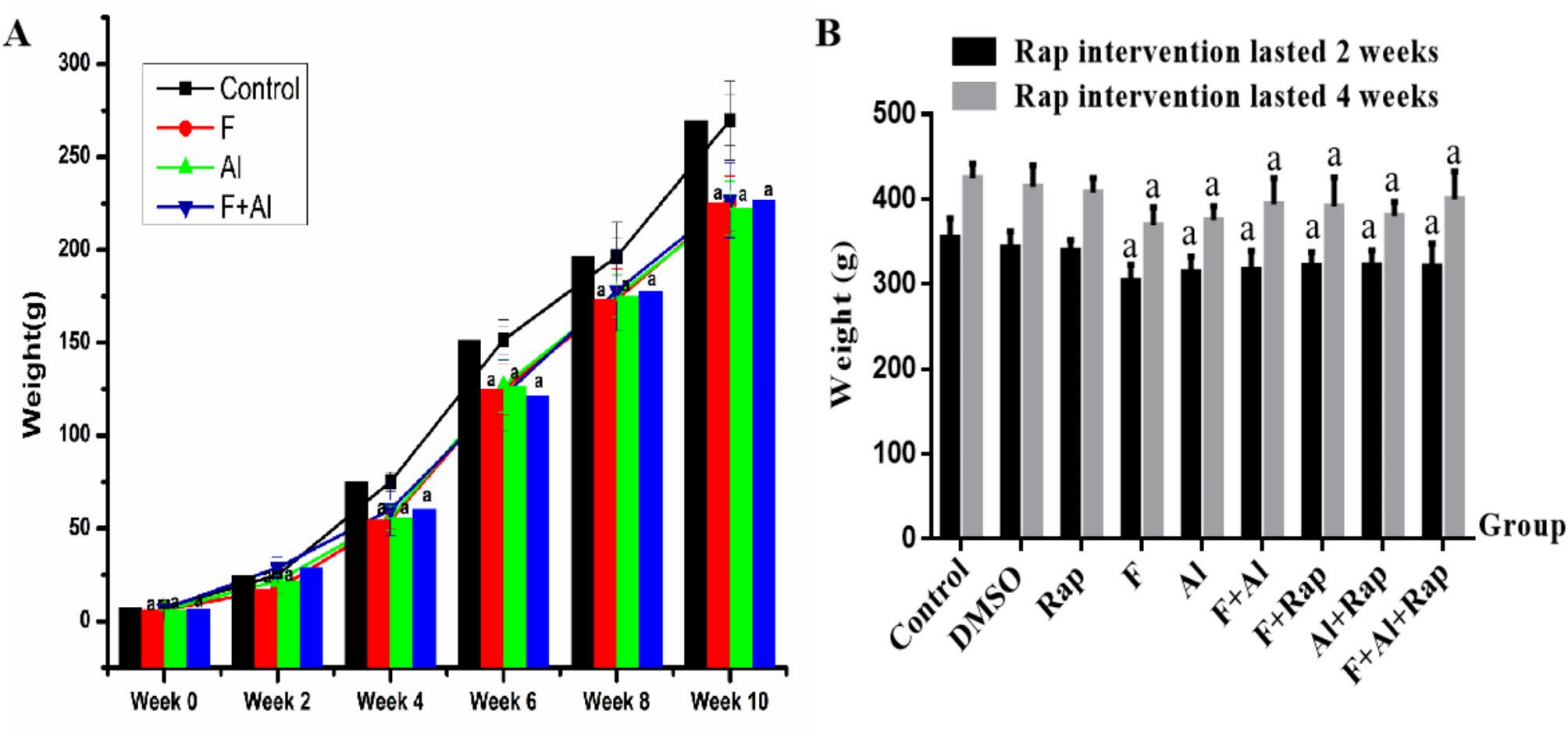
The body weight changes of F2 rat exposed to F, Al and F + Al (**A**). The therapeutic effects of Rap for 2 weeks and 4 weeks on body weight of F2 rat exposed to F, Al and F + Al (**B**). ^a^*P* < 0.05 versus the control group. (*n* = 6).

Figure 4A demonstrated that the body weight of F2 rats in the F + Al group was lower than the control group at 0th, 4th, 6th, 8th and 10th week (*P*<0.05). The weights of F2 rats of F and Al groups were lower than the control group at 2nd week (*P*<0.05).

In Fig. 4B, the body weight of the F2 rats in the experimental groups of Rap treatment lasted for 2 weeks and 4 weeks were lower than the control group (*P*<0.05). However, compared to the F, Al, and F + Al groups, there were no significant change in the body weight of the F2 rats in the Rap, F + Rap, Al + Rap and F + Al + Rap groups.

### The effect of Rap on hippocampal neuron morphology of F2

**Fig. 5:**
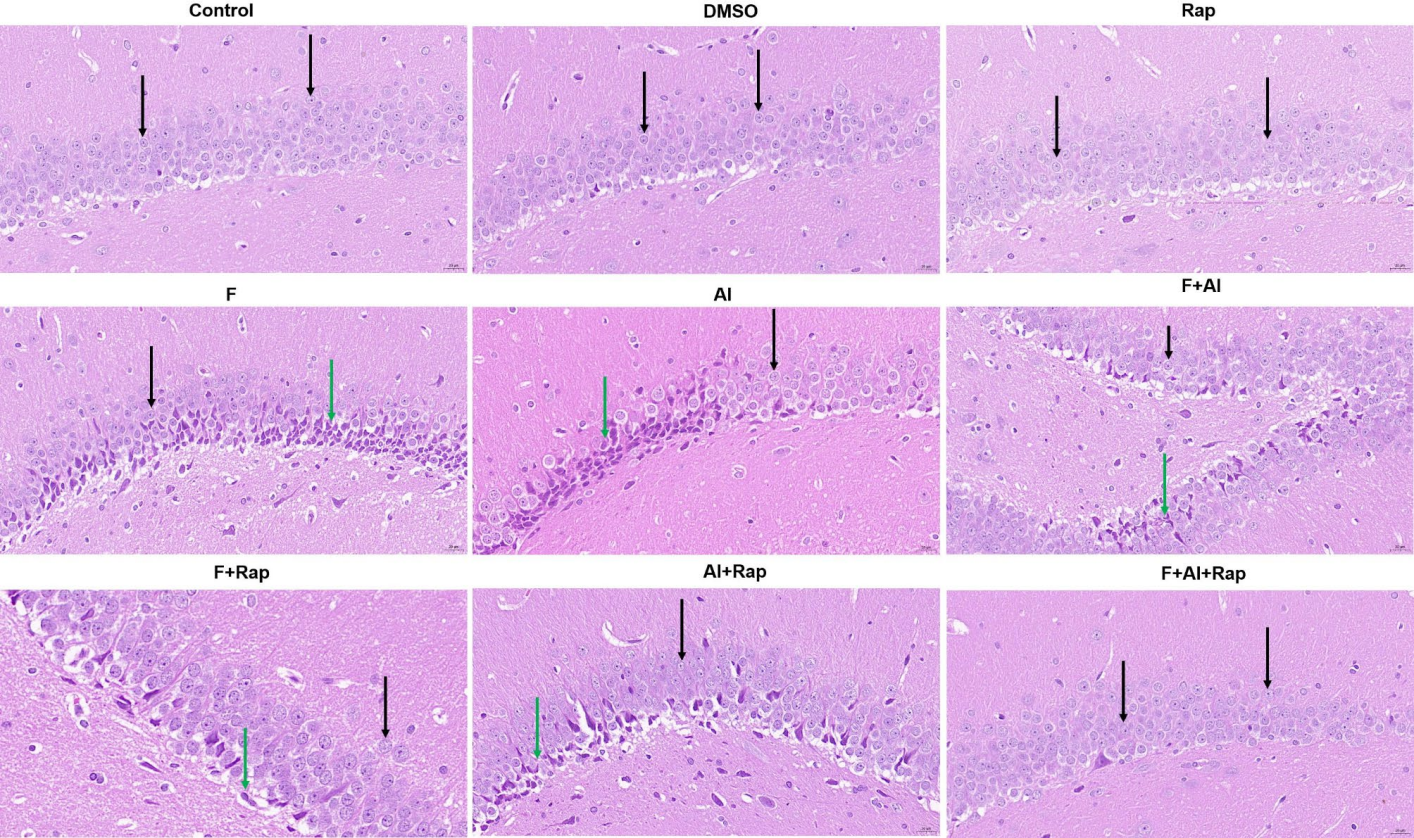
The effects of Rap on the hippocampal structure of the F2 rat exposed to fluorine combined with aluminum by HE staining (×400). The black arrow indicated the normal cells and the green arrow indicated the abnormal cells.

The hippocampal HE staining of F2 rats revealed that the pyramidal cells in the control, DMSO, and Rap groups exhibited regular morphology, uniform light blue staining, clear nucleoli, and abundant cytoplasm, with no observable damage or glial cell proliferation (Fig. 5). In the F group, pyramidal cells in the CA1, CA2, and DG regions of the hippocampus showed constriction, reduced volume, deepened staining, enhanced basophilia, and unclear cytoplasmic and nuclear boundaries. The Al group showed similar changes to the F group, with a small amount of pyramidal cell body compaction observed in the CA1 and DG regions. In the F + Al group, a very small amount of pyramidal cell body compaction was observed in the DG region, with other changes resembling those in the F group. Compared to the exposure groups, some pathological changes were still observed in the F + Rap and Al + Rap groups. However, the pyramidal cells in the hippocampus of the F + Al + Rap group had largely returned to normal, with no obvious damage.

### The effect of Rap on hippocampal ultrastructure of F2 rats exposed to FA

**Fig. 6:**
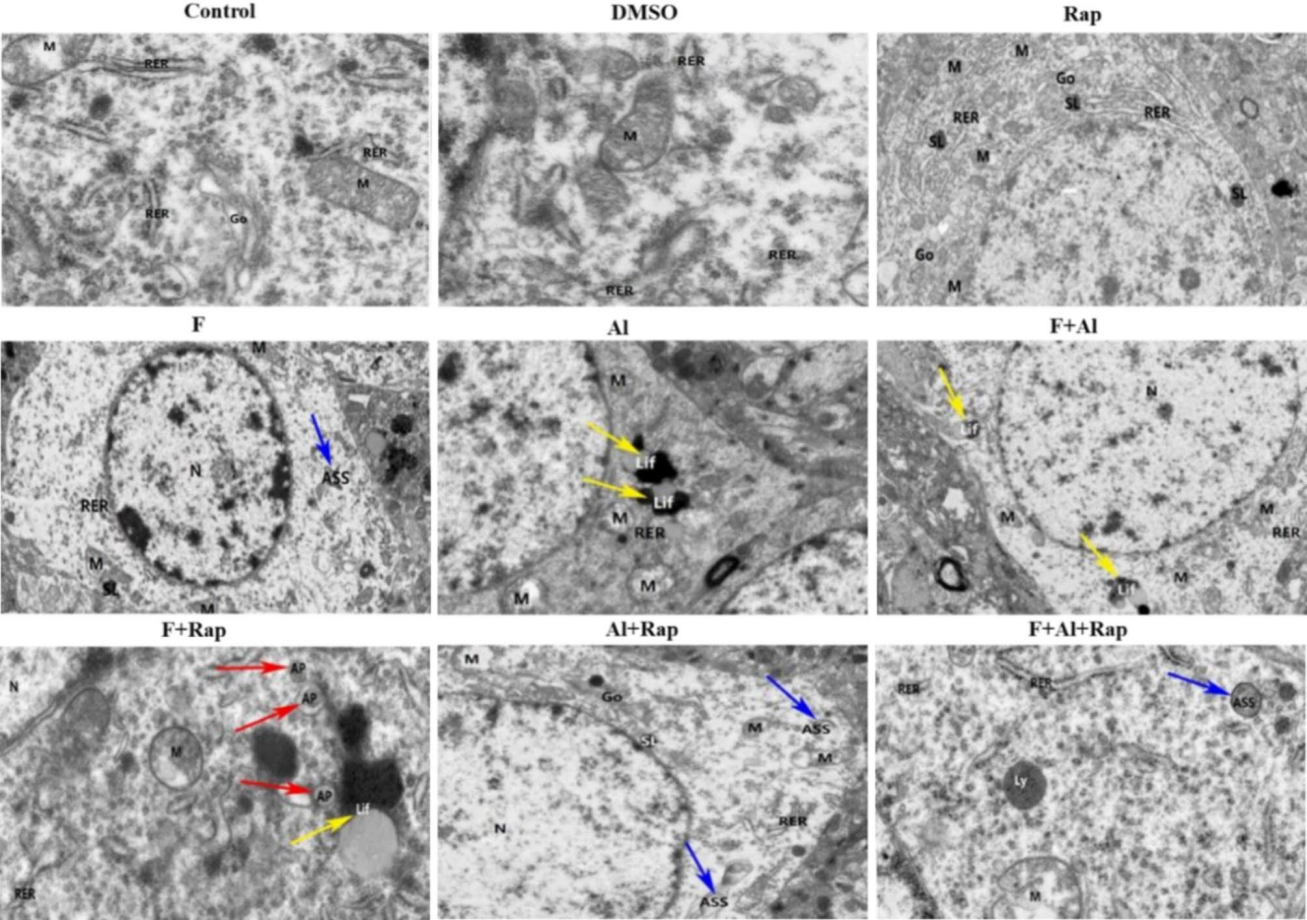
The effects of Rap on hippocampal structure of the F2 rat exposed to FA by TEM (×6000). The red arrow refers to autophagosomes, the blue arrow indicates the autophagic lysosomes, the yellow arrow indicates the lipofuscin (autophagic lysosome residue). N: cell nucleus, M: mitochondria, RER: rough endoplasmic reticulum, Go: Golgi apparatus, SL: secondary lysosomes, AP: autophagosomes, ASS: autophagy lysosome, Lif: lipofuscin, Ly: primary lysosome.

The hippocampal ultrastructures of F2 rats were recorded by TEM, as shown in Fig. 6. In the control group, the neuronal cell cytoplasm displayed slight edema, abundant organelles, and evenly distributed mitochondria with mild swelling and a small number of neatly arranged broken or shortened cristae. The Golgi apparatus membrane was slightly dilated, with no obvious autophagy observed. In the DMSO group, neuronal cells had moderately swollen cytoplasm, reduced organelles, and slightly swollen mitochondria with a shallow and uneven matrix. The rough endoplasmic reticulum showed slight dilation with local degranulation and no obvious autophagy. In the Rap group, neuronal cells exhibited slight edema and abundant organelles, with mitochondria slightly swollen and an occasionally dilated matrix. Some mitochondria were moderately swollen with a shallow and uneven matrix, and partially broken and missing cristae. The rough endoplasmic reticulum showed slight expansion and a small amount of degranulation, with mild dilation of the Golgi capsule membrane. Secondary lysosomes were present inside the cell.

In the F group, neurons exhibited severe edema, sparse cytoplasm, reduced organelles, blurred nuclear membranes, sparse chromatin, and a small amount of heterochromatin border collection. Additionally, there was local swelling and membrane protrusion of individual mitochondria, slight dilation of the rough endoplasmic reticulum, and the presence of autophagic lysosomes. In the Al group, there was no cytoplasmic swelling, local blurring of the nuclear membrane, moderate swelling of mitochondria, large volume, and extensive cristae breakage and dissolution. Lipofuscin was present inside the cell. The F + Al group showed severe cytoplasmic edema, low cytoplasmic electron density, severely swollen and vacuolated mitochondria, slightly expanded rough endoplasmic reticulum with a small amount of degranulation, and the presence of lipofuscin inside the cell.

In the F + Rap group, compared to the F group, there was no obvious swelling of neurons. Half of the mitochondria were severely swollen with partially dissolved, deficient, vacuolated, and uneven matrices, and slightly broken cristae. The rough endoplasmic reticulum appeared in a long linear shape, with a certain amount of lipofuscin and autophagosomes inside the cells. The Al + Rap group showed severe cytoplasmic edema, low cytoplasmic electron density, severely swollen mitochondria with broken and missing cristae, shallow matrices, and increased vacuolated mitochondria volume. Some membrane damage was observed, with partially blurred Golgi body capsule membranes and a small number of secondary lysosomes and autophagic lysosomes. In the F + Al + Rap group, neurons showed mild cytoplasmic swelling, with most mitochondria slightly swollen and shallow matrices. Cristae were reasonably arranged, and a few mitochondria were moderately or severely swollen with enlarged volumes, dissolved matrices, and occasionally damaged membranes. The membranes of the Golgi body were arranged in parallel and slightly dilated locally. A small number of primary lysosomes and autophagic lysosomes were found in the cells.

### The effect of Rap on mRNA and protein expression levels of AMPK/mTOR/ULK1 pathway and LC3 in the hippocampus of F2 rats

**Fig. 7:**
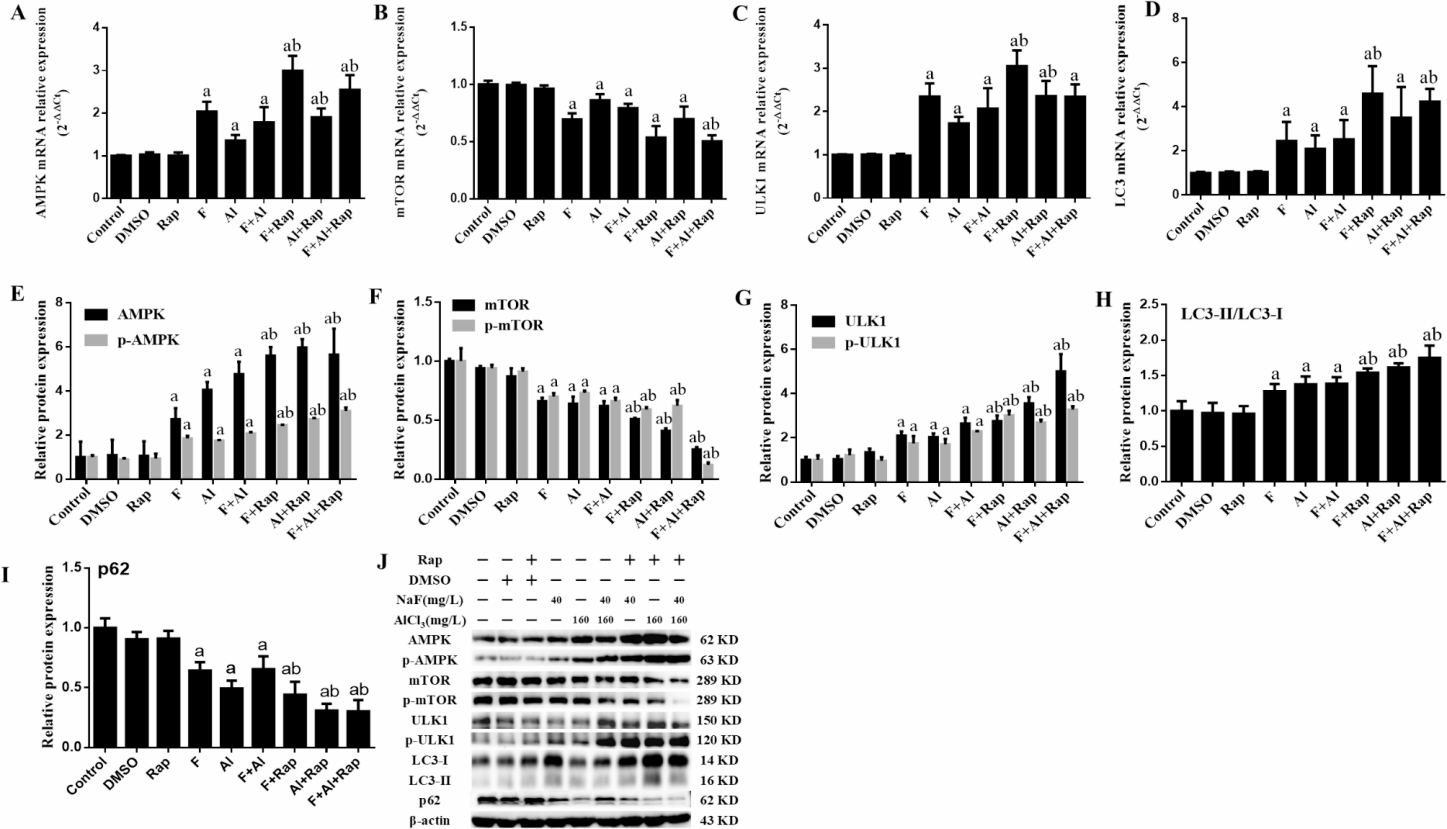
The effects of Rap on the mRNA expression (**A**–**D**) and the protein expression (**E**–**I**) levels of AMPK/mTOR/ULK1 pathway, LC3 and p62 in the hippocampus of F2 rats. The effects of Rap on the original gel electrophoresis of AMPK/mTOR/ULK1 pathway and LC3 protein expression in the hippocampus of F2 rats (**J**). ^a^*P* < 0.05 versus the control group, ^b^*P* < 0.05 versus the exposure groups (*n* = 6).

As shown in Fig. 7A–D, the mRNA expression levels of AMPK, ULK1 and LC3 were significantly higher in the F, Al, F + Al, F + Rap, Al + Rap, and F + Al + Rap groups compared to the control group (*P*<0.05), while the mRNA expression level of mTOR decreased (*P*<0.05). In comparison with the exposure groups, the mRNA expression of AMPK increased significantly in the F + Rap, Al + Rap, and F + Al + Rap groups (*P*<0.05). The mRNA expression of mTOR decreased in the F + Al + Rap group (*P*<0.05), and the mRNA expression of ULK1 increased in the F + Rap and Al + Rap group. Additionally, the mRNA expression of LC3 increased in the F + Rap and the F + Al + Rap groups (*P*<0.05).

The protein expressions of AMPK/mTOR/ULK1 pathway and LC3 was showed in Fig. 7E–J. The protein levels of AMPK, p-AMPK, ULK1 and p-ULK1 and the ratio of LC3-II/LC3-I increased significantly in all experimental groups (*P*<0.05), while the protein levels of mTOR, p-mTOR and p62 decreased (*P*<0.05). Compared to the exposure groups, the protein expressions of AMPK, p-AMPK, ULK1, p-ULK1 and the ratio of LC3-II/LC3-I increased in the F + Rap, Al + Rap, and F + Al + Rap groups, and the protein levels of mTOR, p-mTOR and p62 decreased (*P*<0.05).

## Discussion

In this work, we established the NG108-15 cell model and F2 rat model of FA- exposure with or without Rap treatment. The changes in the hippocampal tissue structure of F2 rats were observed, the autophagy in hippocampal of F2 rats and NG108-15 cells were measured and compared, and the differences in mRNA and protein expression levels of AMPK, mTOR, ULK1, and LC3 were analyzed. The impact of AMPK/mTOR/ULK1 signaling pathway-mediated autophagy in FA on nerve cells as well as the therapeutic effect of Rap were examined.

Prior to measuring the impact of Rap on the viability of NG108-15 cells, our research group has evaluated the effects of F and Al exposure on the survival rate of NG108-15 cells. Both F and Al exposure were found to decrease the survival rate of these cells^36^. In previous studies conducted by our group, the fluoride ion-selective electrode method was employed to determine fluoride content in urine and brain tissue, while inductively coupled plasma optical emission spectrometry was used to measure aluminum content in urine and brain tissue. The detected levels of both elements were increased in the urine and brain tissue of rats exposed to F2 across all exposure groups. These findings indicated that the established cellular and animal models can be used to study the therapeutic effects of Rap.

Changes in body weight can reflect growth and development as well as health status. In this study, the body weight of F2 rats decreased in the exposure groups, indicating that fluorine and aluminum are harmful to body growth. However, there was no significant change in body weight following Rap treatment. Similarly, in the AD transgenic mouse model, Rap did not significantly affect the body weight of AD mice^37^. This suggests that Rap, as a therapeutic agent for neurological diseases, has not exhibited any adverse effects on growth and development indicators, such as body weight.

The normal structure of hippocampal tissue is essential for its function. Previous research has shown that hippocampal neurons are damaged, and their morphological structure is disrupted in ischemic stroke rat models^38^. In this study, HE staining of the hippocampus revealed that pyramidal cells in the CA1, CA2, and DG regions exhibited contracted cell bodies, decreased volume, deepened staining, enhanced basophilia, and unclear cytoplasmic-nuclear boundaries in the exposure groups. Consequently, fluorine and aluminum disrupted the normal hippocampal tissue structure in F2 rats, which may explain the observed impairment in learning and memory.

In the AD rat model, hippocampal neurons structure in the control group was normal, whereas neurons in the CA area of the AD model group were lost, with loosely arranged pyramidal cells and blurred, swollen nuclei. However, after the Rap treatment, neurons morphology was mostly normal, and hippocampal tissue pathology was improved^39^. In this study, some pathological changes were still observed in the hippocampus of the F + Rap and Al + Rap groups after the Rap treatment. However, the morphology of pyramidal cells in the hippocampus of the F + Al + Rap group returned to normal, with no significant damage or glial cell proliferation. In mouse models of brain aging, neurons in the hippocampus of the model group exhibited disordered arrangement and sparse distribution, whereas no obvious morphological damage was observed in the hippocampal neurons after Rap treatment^40^. These findings indicated that Rap could improve can mitigate hippocampal neuron damage caused by combined fluorine and aluminum exposure, exerting neuroprotective effects.

Autophagy is the process essential for cellular metabolism and the renewal of certain organelles. Autophagosomes are responsible for enclosing and transporting substances destined for degradation to lysosomes, forming autophagolysosomes. When the autophagy process is impeded or lysosomal function declines, incompletely digested organelles and macromolecules may accumulate and convert into lipofuscin. The accumulation of lipofuscin can lead to further decline in lysosomal function, thereby affecting the formation and degradation efficiency of autophagosomes. Rap is a classic autophagy agonist that activates the autophagy by inhibiting the synthesis of mTOR, playing a neuroprotective role in various neurodegenerative diseases, such as AD and autism^41^. Previous studies have shown that Rap can regulate the autophagy-related pathways and improve cognition that delayed the progression of neurodegenerative diseases. In the hippocampus, TEM observations revealed decreased cellular organelles and the presence of autophagic lysosomes in the exposure groups, suggesting that fluorine and aluminum can induce autophagy. Moreover, a significant number of autophagosomes and autophagy lysosomes were observed in the cells of all RAP treatment groups, confirming that Rap enhanced autophagy, consistent with previous results^42^. This study further indicated that Rap can amplify the autophagy induced by FA. In the NG108-15 cell, green fluorescent spots were observed in the cytoplasm of the exposure group, and these spots increased in both intensity and volume in the treatment group. This suggests that Rap can further promote the autophagy activity by FA in NG108-15 cells.

AMPK, mTOR and ULK1 are crucial molecular targets of the autophagy initiation, with the AMPK/mTOR/ULK1 signaling pathway playing a significant role in regulating autophagy. AMPK enhances autophagy by inhibiting mTOR activation and directly phosphorylating ULK1, thereby promoting autophagy initiation^27^. LC3, a specific autophagy marker, is involved in autophagosome formation, with the conversion of LC3-I to LC3-II indicating autophagy occurrence^43^. P62 plays a crucial role in autophagy, serving not only as a receptor for selective autophagy but also regulating autophagic activity through its expression levels and modification states^44^. In the animal studies, exposure to fluorine and aluminum resulted in increased protein expression of AMPK, p-AMPK, ULK1, p-ULK1, and the LC3-II/LC3-I ratio, while mTOR, p-mTOR and p62 expression decreased. This suggests that AMPK activation promotes cell autophagy through various pathways when exposed to F and Al^45,46^.

The protein expression levels of AMPK, p-AMPK, ULK1, p-ULK1 and LC3-II/LC3-I were higher in the F + Rap, Al + Rap and F + Al + Rap groups compared to the exposed groups, while the expressions of mTOR, p-mTOR and p62 were lower. This suggested that Rap can further induce and promote the autophagy by up-regulating the AMPK/mTOR/ULK1 signaling pathway and LC3. In the cell studies, the protein levels of AMPK, p-AMPK, ULK1, p-ULK1 and LC3-II/LC3-I ratio increased in the exposed groups, whereas the protein levels of mTOR and p-mTOR decreased in the F and the F + Al groups, the protein levels of p62 decreased in the F, Al and the F + Al groups. These results indicated that FA combined with AMPK activation increases the expression level of p-AMPK, inhibits mTOR activity, and induces autophagy. After Rap treatment, the protein levels of AMPK, p-AMPK, ULK1, p-ULK1 and LC3-II/LC3-I ratio in the F + Rap, Al + Rap and F + Al + Rap groups were higher than in the exposed groups, while the expression of p-mTOR and p62 protein was lower. The expression of mTOR and p62 is suppressed by Rap, leading to a reduction in the binding of mTOR to AMPK and a relative elevation in AMPK expression. This suggests that Rap can further enhance the autophagy induced by F and Al in NG108-15 cells through the AMPK/mTOR/ULK1 pathway.

## Conclusion

In conclusion, the NG108-15 cell model and the F2 rat model of FA exposure with or without Rap treatment were successfully established. It was observed that combined exposure to fluorine and aluminum induced autophagy in hippocampal neurons of F2 rats and NG108-15 cells, which was further enhanced by Rap treatment. Rap treatment reduced hippocampal neuronal damage in F2 rats exposed to FA. Rap enhances FA-induced autophagy by activating the AMPK/mTOR/ULK1 signaling pathway in neurons, thereby ameliorating nerve damage. Thus, Rap may be considered a therapeutic agent for hippocampal neuronal damage caused by FA.

## Materials and methods

### Chemicals and instruments

NG108-15 cells were purchased from Shanghai ATCC Cell Bank; NaF (> 99%) and AlCl_3_ (> 99%) were obtained from Sigma-Aldrich; Rap was provided by Beijing Solarbio Life Science Co., Ltd. The AMPK, mTOR, p-mTOR, ULK1, LC3 and p62 antibodies were sourced from Wuhan proteintech Biotechnology Co., Ltd. The p-AMPK, p-ULK, and β-actin antibodies were acquired from Qingdao Affinity Biotechnology Co., Ltd. CFX96 quantitative real-time fluorescent PCR Instrument (qRT PCR, Bio-Rad Laboratories, Inc, USA), HT7700 transmission electron microscope (TEM, Hitachi High tech Company), Nikon Eclipse E100 upright optical microscope (Nikon Corporation, Japan), Nikon DS-U3 imaging system (Nikon Corporation, Japan), XRS275000 gel imaging system (Bio-Rad Laboratories, Inc, USA), and Nikon TE2000-U inverted fluorescence microscope (Nikon Corporation, Japan).

### Cell treatment

The treating concentrations of NaF and AlCl_3_ in the cell culture medium were 40 mg/L (1/2 IC_50_) and 160 mg/L (1/2 IC_50_), respectively, consistent with our previous study^36^. The experimental groups are detailed in Table S1. Results from the CCK-8 experiment showed that treating NG108-15 cells with 20 µmol/L Rap for 24 h, maintained cell viability above 90%, indicating a significant therapeutic effect. Therefore, this concentration was selected for subsequent experiments. NG108-15 cells were cultured in DMEM medium at 37 °C with 5% CO_2_ in a humidified atmosphere.

### Animal treatments

This experiment was approved by the Ethics Committee of Guizhou Medical University (GMU), with approving number as 1800241(see ARRIVE). The 16 SD pregnant rats (weight 220–250 g) were purchased from the Center of Laboratory Animals of GMU and the facility qualification certificate number was SYXK (Qian) 2018-0001, and the animal quality certificate number was SCXK (Qian) 2018-0001.The pregnant rats were singly housed in an appropriate environment with free access to drinks and food. Based on toxicological design principles and our previous study^47^, the exposure dose of NaF for rats was 120 mg/L (1/10 LD_50)_. Considering the allowable intake of aluminum in food and the safety factor between humans and animals, the exposure dose of AlCl_3_ was set at 600 mg/L (1/10 LD_50_).

16 pregnant rats were randomly divided into four groups based on body weight: one control group and three exposure groups (F, Al, and FA). The NaF and AlCl_3_ concentrations in the drinking water for each group were 0 and 0 mg/L, 120 and 0 mg/L, 0 and 600 mg/L, and 120 and 600 mg/L, respectively. The maternal rats were treated with FA from the beginning of pregnancy until postnatal day 21 (PND21). The first-generation (F1) offspring rats in each group continued to be exposed in the same manner until sexual maturity (PND90). Four female and two male F1 rats were randomly selected from each group and caged. The female F1 rats continued to be exposed until PND21 in the second generation. Eight F2 rats from each group were exposed to FA until the 10th week after birth. The rats’ weights were measured every two weeks. F2 rats were anesthetized with isoflurane and euthanized by collecting blood from the apex of the heart. The right brain tissue was placed in 4% formaldehyde for Hematoxylin-Eosin (HE) staining to observe the morphological changes of hippocampal neurons, or fixed with 2.5% glutaraldehyde for transmission electron microscopy (TEM) to observe the autophagy of the hippocampus. The left-brain tissue was isolated from the hippocampus to extract AMPK, mTOR, ULK1, LC3 and p62 proteins and mRNA. All stored at −80℃ for later use. After F2 rats exhibited neurobehavioral alterations, one F2 rat was randomly dissected from each exposure group to confirm that the exposed substance had caused hippocampal neuronal damage. Subsequently, the remaining F2 rats in each exposure group were administered rapamycin (5 mg/kg) intraperitoneally for seven consecutive days. The experimental groups are detailed in Table S2.

### Autophagy assay

#### Detection of the autophagy in NG108-15 cells using immunofluorescence/immunocytochemistry (IF/ICC) kit

NG108-15 cells were fixed on 24-well plates and incubated with the primary antibody for 1.5 h at room temperature. After washing with PBS, the cells were incubated with fluorescein isothiocyanate-labeled IgG antibody at 37 °C for 1 h in the dark. The cells were then stained with 4’,6-diamidino-2-phenylindole dihydrochloride and observed under inverted fluorescence microscope.

#### Observation of the pathological changes in the hippocampus using HE staining

The hippocampus tissues of F2 rats were removed and fixed in a fixative solution for 24 h. They were dehydrated in 75%, 85%, and 90% alcohol, anhydrous ethanol, alcohol benzene, and xylene solutions, and then immersed in paraffin wax. The wax-soaked tissues were then embedded, and sliced after cooling, with a thickness at 3–5 μm. The floating tissues were picked up by slides and dried at 60℃ for 1 h, and then dewaxed in xylene, anhydrous ethanol and 75% alcohol in turn. The tissues were stained with hematoxylin and eosin. Following a second dehydration, the tissues were sealed with neutral resin and examined under upright fluorescence microscope at Wuhan Servicebio Co., Ltd.

#### Observation of the autophagy in hippocampus of F2 rats using TEM

1 mm³ sample of hippocampus tissue was fixed in electron microscope fixation solution for 4 h. After rinsing with PBS, the tissues were dehydrated with 50%, 70%, 80%, 90%, 95%, 100% alcohol, then permeated and embedded overnight with acetone and 812 embedding agents at 37 ℃, sectioned into 60–80 nm ultra-thin slices, and stained with 2% uranium acetate saturated alcohol solution and 2.6% lead citrate solution, and finally observed under TEM at Wuhan Servicebio Co., Ltd.

### Detection of RNA expression using qRTPCR

Based on our previous reports, Trizol reagent was used to extract RNA from tissue and cells^13,36^. The forward and reverse primer sequences of AMPK, mTOR, ULK1, LC3 and GAPDH are listed in Table S3. Their levels were measured using qRTPCR, and quantified using the 2^−ΔΔCt^ method.

### Detection of protein expression using western blotting (WB)

Proteins from the hippocampus and cells were extracted using lysis buffer containing 1% PMSF protease inhibitor and quantified using the BCA assay. The electrophoresis and membrane transfer procedures followed those described in our previous reports^13,36^. Select the separation gel and 5% concentrated gel corresponding to the molecular weight of the target protein for electrophoresis. Polyvinylidene difluoride membranes were incubated overnight at 4 °C with the primary antibodies against β-actin, AMPK, p-AMPK, mTOR, p-mTOR, ULK1, p-ULK, LC3 and p62. This was followed by incubation with appropriate secondary antibodies for 1.5 h at room temperature and visualization using an ECL kit. To prevent interference from excessive nonspecific bands, the blots of the target proteins were cut out prior to hybridization with the antibodies. Chemiluminescence images were analyzed with Image J.

### Data analyses

Statistical analyses were conducted using SPSS (Version 22.0, Chicago, IL, USA). Data are presented as the means ± standard deviations (Mean ± SD). One-way analysis of variance (ANOVA), along with the LSD and Dunnett’s T3 test method, were used to analyze the experimental data. A *P* value < 0.05 was defined as statistically significant.

## Electronic supplementary material

Below is the link to the electronic supplementary material.

Supplementary Material 1

## Data Availability

Data could be provided if required. If anyone wishes to request data from this study, please contact the corresponding author Prof. Chun Xie (email: 1009207189@qq.com ).

## Declarations

### Animal experiment statement

This study has been approved by the Ethics Committee of Guizhou Medical University with approval number 1800241. The experiment is performed in accordance with the relevant guidelines and regulations of GMU animal experiment. All methods are reported in accordance with the ARRIVE Guide.
