# Supplementary material for: Rapamycin mitigates neurotoxicity of fluoride and aluminum by activating autophagy through the AMPK/mTOR/ULK1 pathway in hippocampal neurons and NG108-15 cells
Source: Sci Rep. 2025 Mar 21;15:9801. doi: 10.1038/s41598-025-94648-0 (PMC11928598; doi:10.1038/s41598-025-94648-0)
Supplement: Supplementary file 1 — Supplementary Material 1 [file 41598_2025_94648_MOESM1_ESM.pdf]

Rapamycin activates autophagy via the AMPK/mTOR/ULK1 signaling pathway to mitigate the neurotoxic effects of fluoride combined with aluminum on hippocampal neurons and NG108-15 cells

Dan Tao[1, #], Ya Xia[1, #],Qilong Liao[2, #], Xuemei Yang[1], Luwen Zhang[1], Chun Xie[1, *]

[1] School of Public Health, the key Laboratory of Environmental Pollution Monitoring and Disease Control, Ministry of Education, Guizhou Medical University, No.6 Ankang Road, Guian New Area, Guizhou, 561113, China

[2] State Environmental Protection Key Laboratory of Environmental Pollution Health Risk Assessment, Research Center of Emerging Contaminants, South China Institute of Environmental Sciences, Ministry of Ecology and Environment, Guangzhou 510655, PR China.

[#] These authors contributed equally to this work.

*Corresponding author:

Chun Xie. School of Public Health, Guizhou Medical University, No.6 Ankang Road, Guian New Area, Guizhou, China

E-mail address: 1009207189@qq.com

Table S1 Different treatment groups of NG108-15 cells

Groups	NaF（mg/L）	$AlCl_3$（mg/L）	Rap（μmol/L）
Control	0	0	0
DMSO	0	0	0
Rap	0	0	20
F	40	0	0
Al	0	160	0
F+Al	40	160	0
F+Rap	40	0	20
Al+Rap	0	160	20
F+Al+Rap	40	160	20

Table S2 Different treatment groups of rats

Groups	NaF（mg/L）	$AlCl_3$（mg/L）	Rap（mg/kg）
Control	0	0	0
DMSO	0	0	0
Rap	0	0	5
F	120	0	0
Al	0	600	0
F+Al	120	600	0
F+Rap	120	0	5
Al+Rap	0	600	5
F+Al+Rap	120	600	5

Table S3 the primer sequences of AMPK, mTOR, ULK1, LC3 and GAPDH

Gene	Primer sequence	
	Forward	Reverse
AMPK	5’-GTC AAA GCC GAC CCA ATG ATA-3’	5’-CGT ACA CGC AAA TAA TAG GGG TT-3’
mTOR	5’-GGC ACA CAT TTG AAG AAG CAG-3’	5’-CTC GTT GAG GAT CAG CAA GG-3’
ULK1	5’-ACA TCC GAG TCA AGA TTG CTG-3’	5’-GCT GGG ACA TAA TGA CCT CAG G-3’
LC3	5’-GAC CGC TGT AAG GAG GTG C-3’	5’-CTT GAC CAA CTC GCT CAT GTT A-3’
GAPDH	5’-AAG AAG GTG GTG AAG CAG G-3’	5’-GAA GGT GGA AGA GTG GGA GT-3’
